# Optimization of a Tracking-Based Approach for Calculating Energy Expenditure and Aerobic–Anaerobic Supplies During Intermittent Running: Improved Simulation of Oxygen Uptake Within the Metabolic Power Model

**DOI:** 10.3390/s25247568

**Published:** 2025-12-12

**Authors:** Joana Brochhagen, Tjorven Schnack, Christian Baumgart, Matthias W. Hoppe

**Affiliations:** 1 Exercise Science, Institute of Sport Science and Motology, Marburg University, 35032 Marburg, Germany; matthias.hoppe@uni-marburg.de; 2 Department of Sport and Human Movement Science, Centre for Sport Science and University Sports, University of Vienna, 1150 Vienna, Austria; tjorven.josef.schnack@univie.ac.at; 3 Vienna Doctoral School of Pharmaceutical, Nutritional and Sport Sciences, University of Vienna, 1090 Vienna, Austria; 4 Department of Movement and Training Science, University of Wuppertal, 42119 Wuppertal, Germany; baumgart@uni-wuppertal.de

**Keywords:** dynamical analysis, energy cost, global positioning system, PCr-La-O2-model, team sports

## Abstract

**Highlights:**

**What are the main findings?**

- Optimization of the simulated oxygen uptake alone still resulted in both large overestimations and underestimations of total energy expenditure and aerobic–anaerobic energy supplies.
- Only when combined with the corrected calculation of the aerobic energy supply, no statistically significant differences were found for total energy expenditure and aerobic supply, except during repeated sprints.

**What are the implication of the main findings?**

- The anaerobic energy supply is still largely overestimated, thus the calculation of the metabolic power itself should be reconsidered.
- Even more precise results concerning the distinction between aerobic–anaerobic energy supplies could be achieved by including physiological data, namely heart rate, into the simulation of oxygen uptake.

**Abstract:**

In intermittent sports, tracking technologies are commonly used to monitor external and internal loads. The metabolic power model solely uses speed and acceleration data to simulate metabolic power, oxygen uptake, energy expenditure, and aerobic–anaerobic supplies. This study aimed to improve the simulation of oxygen uptake within the metabolic power model, thereby increasing its validity to estimate metabolic loads during intermittent running. Twelve male athletes (24 ± 3 years) performed different intermittent running-based exercises. These data were previously collected and used for secondary analysis within this study. The simulation of oxygen uptake was optimized by different approaches: (i) formerly detected bias (Offset model), (ii) data-driven modeling using differential evolution (Mongin model), and (iii) correction of the aerobic supply calculation. The simulations were compared to the measured oxygen uptake via a portable respiratory gas analyzer and the resulting metabolic loads to those derived by the established 3-component model. For statistical analysis, one-way repeated measures ANOVA or Friedman test with corresponding effect sizes were applied. Overall, the Mongin model demonstrated the best predictive accuracy (MAE = 4.99 ± 1.12 mL/min/kg) compared to measured oxygen uptake and, combined with the corrected calculation, total energy expenditure and aerobic supply did not significantly differ to the standard (*p* ≥ 0.056; trivial to large effect sizes). In conclusion, our optimizations reduce discrepancies of the tracking-based metabolic power model regarding total energy expenditure and aerobic supply compared to the established 3-component model.

## 1. Introduction

The monitoring of external and internal loads during the entire training process is fundamental for optimizing adaptation processes in intermittent sports [1,2]. Over the last decades, access to external loads such as speed and acceleration during training and competition has become standard by tracking technologies, e.g., global and local positioning systems [3,4]. Of note, such technologies also allow to estimate internal loads with respect to energy metabolism, which usually requires invasive capillary blood techniques and non-practical respiratory gas analyses [5]. One mathematical approach using these advances is the metabolic power model. The model calculates metabolic loads, i.e., energy expenditure and aerobic–anaerobic supplies, using only speed and acceleration data via an equivalent slope approach of acceleration and energy costs [5,6]. Even though the potential of the metabolic power model is promising, its validity for intermittent running is still debatable [7].

Past validation studies showed that the metabolic power model underestimates the energy expenditure in intermittent activities when compared to measured oxygen uptake during team-sport-specific circuits by up to 52% [8,9,10]. However, in addition to different running exercises, these circuits also included activities such as collisions [9], ball handling [8], or running backward or sideways [9], which cannot be registered by the metabolic power model. Importantly, intermittent activities are characterized by passive breaks, which the mentioned studies also included in their calculations. The metabolic power model, however, relies on speed and acceleration data, which cannot be generated during these breaks, whereas oxygen uptake is still elevated [5]. Furthermore, measuring oxygen uptake only provides the aerobic energy supply, whereas both aerobic and anaerobic energy supplies are given by the metabolic power model. The distinction between both energy supply pathways is based on the simulation of oxygen uptake in relation to metabolic power within the model [11]. A recent, more thorough validation study [12] took these flaws into account by comparing aerobic–anaerobic energy supplies provided by the metabolic power model to those calculated by an established comparative standard, which is the 3-component model, also known as the PCr-La-O2 model [13], during different running-based exercises. This study revealed that the simulated oxygen uptake by the metabolic power model shows vertically shifted and non-physiological time courses compared to the measured oxygen uptake, resulting in largely underestimated aerobic and simultaneously largely overestimated anaerobic energy supply by the model. Thus, and due to its potential in the field of exercise science and physiology, an optimization of the tracking-based metabolic power model is warranted.

In fact, to obtain a more physiological and valid view on the energy expenditure and distribution of aerobic–anaerobic energy supplies in intermittent sports by the metabolic power model, there is a need to optimize, in particular, the simulated oxygen uptake. For this, different approaches can be implemented. First, the detected vertical shift [12] can be taken into account by correcting the simulated oxygen uptake by the Δ oxygen uptake per minute gathered through the comparison to the measured oxygen uptake. This way, however, the general time course of the simulated oxygen uptake remains unchanged. Since this time course was considered un-physiological [12], another approach may be a completely new simulation of oxygen uptake. This can be achieved by, e.g., machine learning, which has been shown by previous studies [14,15]. Lastly, the calculation of the aerobic energy supply, also impacting the calculation basis of the anaerobic contribution, needs to be reconsidered. In this context, it is worth mentioning that the metabolic power model was originally designed for calculating the energy expenditure of running only [6,16], but may have to be corrected when taking that of the whole body, as indicated by capillary blood lactate or respiratory gas measures, into account.

This study aimed to improve the simulation of oxygen uptake within the metabolic power model, thereby increasing the validity of the model to estimate energy expenditure and aerobic–anaerobic supplies during intermittent running.

## 2. Materials and Methods

### 2.1. Study Design

The data collection for this study was already completed as part of a former validation study [12]. That study explains the following methodological procedures in more detail. The validation study compared the energy supplies and total energy expenditure provided by the metabolic power model and the 3-component model during three intermittent running-based exercises. The present study employed a secondary analysis of the previously published datasets to implement innovative optimization procedures to improve the simulation of oxygen uptake within the metabolic power model, thereby increasing its validity in estimating metabolic loads during intermittent running. Consequently, there is no duplication of the already published data.

Briefly, to optimize the metabolic power model, all measurements were performed outdoors on a regular tartan track on four days separated by one week each. On the first day, resting oxygen uptake was determined by measuring oxygen uptake in a seated position for 10 min [17]. Then, to validly and reliably measure maximum oxygen uptake in a sport-specific manner [18,19], an interval shuttle run test (ISRT) was performed as described before until individual exhaustion [19]. The measured maximum oxygen uptake was later used as a cut-off for the simulation of oxygen uptake during intermittent running. On the second, third, and fourth day, three running-based exercises, intended to primarily stress either the aerobic, anaerobic alactic, or anaerobic lactic energy supply, were performed in a randomized order using a Latin square counterbalancing design. The exercises were preceded by a 10-min standardized warm-up and were as follows: (i) continuous shuttle runs, 20 + 20 m at 8 km/h for 10 min; (ii) repeated accelerations, 6 × 5 + 5 m with 180° change of direction with 2-min recoveries between accelerations; and (iii) repeated sprints, 3 × 4 × 20 + 20 m with 180° change of direction with 20-s recoveries between sprints and 3-min recoveries between sets. During the ISRT and three exercises, oxygen uptake was measured breath-by-breath by a portable respiratory gas analyzer (Cortex Medical, MetaMax 3B, Leipzig, Germany) until seven minutes post-exercise and resting oxygen uptake was subtracted. Capillary blood lactate was also captured until seven minutes post-exercise and analyzed by an electro-enzymatic analyzer (EKF-diagnostics, Biosen C_line Sport, Cardiff, UK). Regarding the three running-based exercises, the metabolic power model and the 3-component model were applied simultaneously. For the former, the necessary data—namely speed and acceleration—was collected by a 20 Hz global positioning system (exelio srl, GPEXE LT, Udine, Italy). Thereby, simulated and measured oxygen uptake, as well as total energy expenditure, aerobic, and anaerobic energy supplies were provided, as described in detail before [11,13]. Regarding the optimization approaches described below, the simulations of oxygen uptake were compared to the measured oxygen uptake via the portable respiratory gas analyzer and the resulting metabolic loads to those derived by the 3-component model. The relative error of the used portable respiratory gas analyzer, reliability (CV) of the lactate analyzer, and typical error of the global positioning system for speed and acceleration measures are reported as 1.6% [20], 1.3% [21], 2.3%, and 5.6% [22], respectively.

### 2.2. Participants

Twelve male athletes (24 ± 3 years; 185.0 ± 9.1 cm; 81.2 ± 11.1 kg) participated. The sample size was chosen based on previous similar validation studies including 10 to 14 participants [8,10,23,24]. All participants were sports students from the local university. Inclusion criteria were males with an age of 18 to 30, who participated three to four times per week in an intermittent team sport. Exclusion criteria were acute or chronic diseases and injuries speaking against maximum testing. All participants signed a written informed consent. The study was approved by the Ethics Committee of Leipzig University (2202.02.23_eb136) and conducted in accordance with the Declaration of Helsinki.

### 2.3. Metabolic Power Model

The used global positioning system provided the speed and acceleration data to calculate metabolic power (Equations (1) and (2)) and subsequently simulate net oxygen uptake (Equation (3)) with the following equations [5,11]:

(1) $$EC=(155.4{ES}^{5}-30.4{ES}^{4}-43.3{ES}^{3}+46.3{ES}^{2}+19.5ES+3.6)\cdot EM$$

(2) P=EC·v

(3) $$\dot{V}O_{2}T_{n(t)}=\left({\dot{E}}_{n}-\dot{V}O_{2}T_{n\left(0\right)}\right)\cdot (1-e^{-\frac{t}{\tau }})+\dot{V}O_{2}T_{\left(0\right)}$$

where *EC* is the energy cost, *ES* is the equivalent slope, 3.6 is the relative energy cost for running at constant speed, *EM* is the equivalent mass, *P* is the metabolic power in W/kg, *v* is the speed, $\dot{V}O_{2}T_{n(t)}$ is the theoretical oxygen uptake at time *t*, $\dot{V}O_{2}T_{\left(0\right)}$ is the theoretical oxygen uptake at the respective onset of each interval, $\dot{E}$ is the metabolic power in equivalent oxygen uptake units, and τ is a time constant. To prevent the simulated oxygen uptake from rising to unphysiologically high levels, the net maximum oxygen uptake measured during the ISRT was used as a cut-off. The model assumes that the aerobic energy supply is the time integral below the course of the simulated oxygen uptake or the course of the metabolic power, if it is below the course of the simulated oxygen uptake. The anaerobic supply is defined as the time integral below the course of the metabolic power, but above the simulated oxygen uptake [11].

### 2.4. 3-Component Model

The 3-component model distinguishes between aerobic, anaerobic alatic, and anaerobic lactic energy supply. The calculations were carried out as described before [13]. Briefly, for the net aerobic energy supply, the resting oxygen uptake, adjusted for the duration of the exercise, was subtracted from the measured oxygen uptake during the exercise [13,17]. For the anaerobic alactic energy supply, oxygen uptake was measured for an additional 7 min after each exercise [17]. Then, the alactic energy supply was calculated from the excess post-exercise oxygen consumption, which was fitted by a bi-exponential function and cut-off at 2 *τ*_a_ to account for the fast component. Again, resting oxygen uptake was subtracted from the oxygen uptake during the fast component [13,17]. The anaerobic lactic energy supply was determined by the Δ lactate for which capillary blood samples were taken from the earlobe before and during the 1st, 3rd, 5th, and 7th minute after each exercise. Then, resting lactate was subtracted from the highest post-exercise lactate. For the lactic energy supply, 1 mmol/L was considered equivalent to 3 mL O_2_/kg [17,25].

### 2.5. Optimization Approaches

The optimization of the metabolic power model regarding the simulation of the oxygen uptake, and thus aerobic and anaerobic energy contributions, was conducted by three different approaches.

First, based on the former validation study [12], the discrepancies of the time courses between measured and simulated oxygen uptake were taken into account. The mean systematic bias of the accumulated oxygen uptake across all participants and all running-based exercises was 0.49 L/min. Therefore, the oxygen uptake simulated by the metabolic power model was initially optimized by exactly this measured offset for each participant and exercise (Offset model).

Second, a more data-driven approach was considered (Mongin model). It has been shown that the relationship between mechanical power and oxygen uptake can be modeled by the following equation [26]:

(4) $$\dot{V}O_{2}^{\prime }\left(t\right)=\frac{K}{\tau }\cdot P\left(t\right)-\frac{\dot{V}O_{2}\left(t\right)-\dot{V}O_{2_{0}}}{\tau }$$

where $\dot{V}O_{2}\left(t\right)$ is the oxygen uptake over time, $\dot{V}O_{2}^{\prime }\left(t\right)$ is its time derivative, $\dot{V}O_{2_{0}}$ is the oxygen uptake at rest, *K* is the proportionality between power and oxygen uptake, τ is the time constant, and *P*(*t*) is the produced power. For this study, the metabolic power, instead of the mechanical power, was used. Since Equation (4) is a differential equation, it was solved numerically for $\dot{V}O_{2}\left(t\right)$ using the SciPy implementation of the Runge–Kutta–Fehlberg method RK45 [27,28]. Differential evolution was used as an optimization algorithm to fit $\dot{V}O_{2_{0}}$, K, and τ to given pairs of power and oxygen uptake [28,29]. The control variables of this algorithm were the number of iterations and population size, which were set to 200 and 20, respectively. These values were considered to be adequate, as higher values did not lead to better results. The goodness of fit was quantified using the coefficient of determination R^2^. To prevent overfitting, the prediction accuracy was assessed in a leave-one-out cross-validation procedure at participant-level: using differential evolution, the optimal values for $\dot{V}O_{2_{0}}$, *K*, and τ were found for the data of all but one participant. Then, these parameter values were employed in Equation (4) to estimate the oxygen uptake of the leftover participant. This process was repeated for each participant. Overall values for $\dot{V}O_{2_{0}}$, *K*, and τ were 0.51 ± 0.03 L/min, 0.21 ± 0.01 (L/min)/(W/kg), and 664.2 ± 35.1 s, respectively.

**Figure 1:**
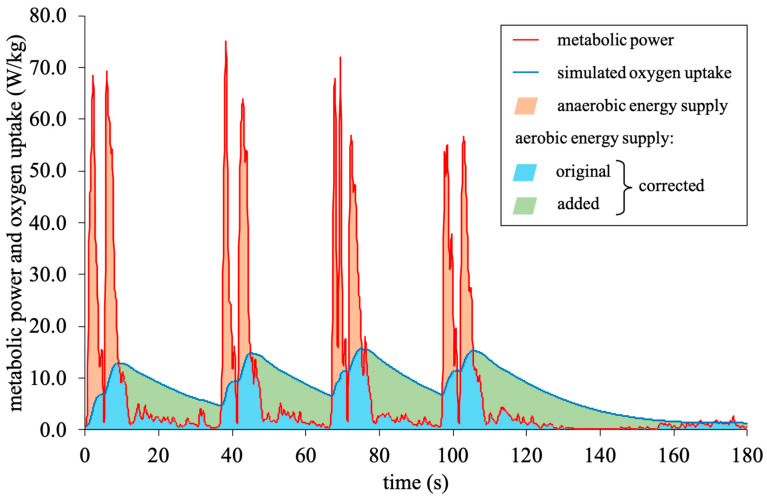
Calculation of the aerobic and anaerobic energy supply by the metabolic power model displaying the corrected aerobic calculation (original + added proportions).

Lastly, and on top of the two former optimization approaches, the calculation of the aerobic energy supply itself was considered. In contrast to the metabolic power model’s assumptions, the complete integral below the course of the simulated oxygen uptake— independent of the time course of metabolic power—was considered (corrected aerobic calculation) (Figure 1). This was due to the original assumption that the model’s calculations may not be appropriate for estimating the overall energy expenditure of exercises (i.e., of the whole body) but rather that of running only, which may not be conclusive enough for training and match analyses [12].

### 2.6. Statistical Analysis

Initially, to quantify prediction accuracy of the simulated oxygen uptake to the measured oxygen uptake, root mean square errors (RMSEs) and mean absolute errors (MAEs) were calculated. For further statistical analysis, data of the energy expenditure and supplies for each running-based exercise were expressed as means ± standard deviations. Normal distribution and variance homogeneity were assessed using Shapiro–Wilk’s and Levene’s test, respectively. Statistical significance was set to *p* < 0.05. If both normality and variance homogeneity were given, one-way repeated measures ANOVA was applied; or else, the Friedman test was used to compare global differences in means. This was decided for each variable individually. Global effect sizes were calculated accordingly, either as generalized eta-squared or Kendall’s W and interpreted as small (≥0.01; <0.3), moderate (≥0.059; <0.5), or large (≥0.138; ≥0.5), respectively. Post hoc pairwise comparisons in means were conducted using *t*-tests or Wilcoxon tests, depending on the normal distribution and variance homogeneity, with Bonferroni correction applied. Pairwise effect sizes were expressed as Cohen’s d and interpreted as trivial (<0.2), small (≥0.2), moderate (≥0.5), or large (≥0.8).

## 3. Results

### 3.1. Simulation of Oxygen Uptake Compared to Measured Oxygen Uptake

**Figure 2:**
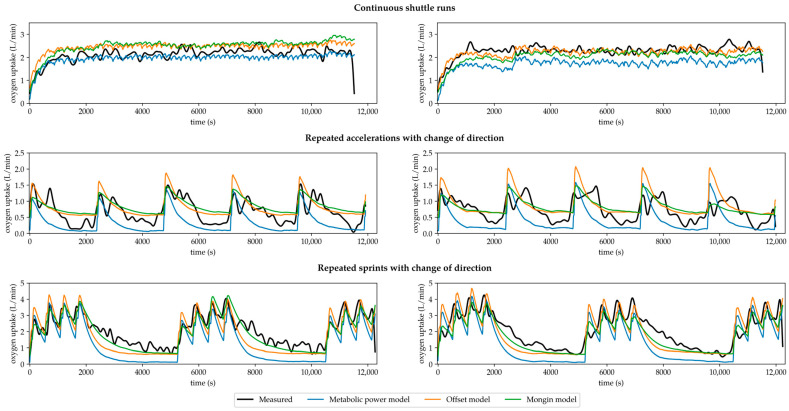
Sample courses of the measured oxygen uptake compared to the simulated oxygen uptakes by the metabolic power, Offset, and Mongin model.

Table 1 displays the prediction accuracy of the simulated oxygen uptake by the metabolic power model as well as by the optimized Offset and Mongin model compared to the measured oxygen uptake. For the continuous shuttle runs, the Offset model had the lowest RMSE (4.19 ± 1.82 mL/min/kg) and MAE (3.60 + 1.92 mL/min/kg). For the repeated accelerations, the Offset and Mongin model achieved similar results with RMSEs of 4.71 ± 0.98 and 4.71 ± 1.04 mL/min/kg and MAEs of 3.82 ± 0.76 and 3.88 ± 0.83 mL/min/kg. For the repeated sprints, the Mongin model showed the lowest RMSE (8.37 ± 1.71 mL/min/kg) and MAE (6.58 ± 1.65 mL/min/kg). For the overall assessment, the Mongin model performed best with an RMSE of 6.05 ± 1.10 and MAE of 4.99 ± 1.12 mL/min/kg. For visualization, Figure 2 shows examples of the measured and three simulated oxygen uptake curves (metabolic power vs. Offset vs. Mongin model) for two randomly selected participants performing the three running-based exercises.

### 3.2. Simulation of Energy Expenditure and Aerobic–Anaerobic Supplies Compared to Those Derived by the 3-Component Model

**Figure 3:**
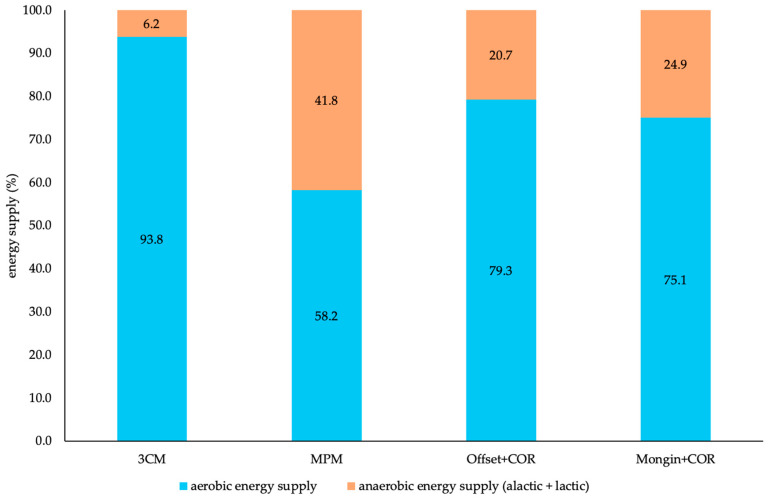
Relative aerobic and anaerobic energy supplies of the 3-component, metabolic power, Offset, and Mongin model for the overall assessment (n = 12). 3CM = 3-component model; COR = corrected aerobic calculation; MPM = metabolic power model.

Table 2 presents the differences in total energy expenditure and aerobic–anaerobic supplies during the three running-based exercises of the 3-component and original metabolic power model as well as optimized Offset and Mongin model. The corrected calculations regarding aerobic energy supply within the metabolic power model are also included. For the continuous shuttle runs, there were no statistically significant differences for the corrected aerobic supply and corrected total energy expenditure between the 3-component model and both the Offset and Mongin model (*p* ≥ 0.840; trivial to moderate), as well as the 3-component model and all three metabolic power, Offset, and Mongin model (*p* ≥ 0.067; small to large), respectively. For the repeated accelerations, there were no statistically significant differences between the corrected aerobic supply and the corrected total energy expenditure between the 3-component and both Offset and Mongin model (*p* ≥ 0.097; trivial to moderate). For the overall perspective, there were no statistically significant differences for the corrected aerobic supply and corrected total energy expenditure between the 3-component and both Offset and Mongin model (*p* ≥ 0.056; small to large), as well as the 3-component and Mongin model (*p* = 0.384; moderate), respectively. All other variables showed statistically significant differences (*p* ≤ 0.040; small to large). Additionally, Figure 3 shows the relative energy supplies concerning the overall perspective for the 3-component and metabolic power model, as well as the Offset and Mongin model, including the corrected aerobic calculation.

## 4. Discussion

This study aimed to improve the simulation of oxygen uptake within the tracking-based metabolic power model, thereby increasing the validity of the model to estimate energy expenditure and aerobic–anaerobic supplies during intermittent running. The main findings were that (i) the two optimization approaches show better prediction accuracies of the simulated oxygen uptake compared to the original metabolic power model, but (ii) continue to largely underestimate or overestimate energy supplies; however, (iii) when combined with the modified interpretation of the calculation of aerobic energy supply, there were no longer statistically significant differences in aerobic energy supplies and total energy expenditures during the continuous shuttle runs, repeated accelerations, and overall assessment for the Mongin model, whereas anaerobic energy supplies still remain overestimated.

Regarding our first main finding, the quantification of the prediction accuracy for the simulation of oxygen uptake showed better results for the two optimization approaches than for the original metabolic power model (Table 1). For the overall assessment, the Mongin model achieved the best results with an MAE of 4.99 ± 1.12 mL/min/kg, performing better than the Offset model and the metabolic power model with 5.25 ± 0.70 and 7.49 ± 1.13 mL/min/kg, respectively. One of only two available previous studies using machine learning approaches considered heart and breathing rate, as well as speed and acceleration data, as inputs for deep learning models to simulate oxygen uptake, showing a best MAE of 3.70 mL/min/kg [15]. This result is comparable to the MAEs of our Offset model with 3.60 ± 1.92 and 3.82 ± 0.76 mL/min/kg for the continuous shuttle runs and repeated accelerations, respectively. Sheridan et al. [15] had their participants perform an intermittent team sport-specific circuit including walking, jogging, sprinting, changes of direction, jumps, and tackles lasting 45 s with a 15 s break, which was repeated five times. In contrast, the running-based exercises of the present study solely focused on running activities without jumps or tackles due to the inability of the metabolic power model to track those activities. Therefore, a direct comparison to the overall assessment of the three running-based exercises would be more fitting, showing that the results by Sheridan et al. [15] outperformed the Mongin model. However, in this context, it is important to notice that Sheridan et al. [15] not only used speed and acceleration as in our study, but also physiological data, namely heart and breathing rate, to simulate oxygen uptake. This may be a reason for the discrepancies between both outcomes. In another study, participants were tested for steady state conditions at four different walking and running speeds for three minutes each [14]. Oxygen uptake was measured by a portable gas analyzer and simulated by machine learning models using heart rate, step speed, and step duration data as input. When only using heart rate, the MAE was 2.52 mL/min/kg and combined with speed and acceleration data, it was 1.36 mL/min/kg, showing better results than the study by Sheridan et al. [15]. However, the discrepancies may be due to the consistent speed and non-intermittent character of the tests. Nevertheless, both studies showed better results when not only mechanical but also physiological data were included, which is not possible using the metabolic power model alone. Therefore, better results concerning the simulation of oxygen uptake may be achieved if biosignals, e.g., the heart rate, were also taken into account. The implementation into practice would be easily accessible as heart rate monitors are often worn alongside global or local positioning systems, especially during training. Thus, future studies should include heart rate data, which in turn can be helpful for a more precise distinction between energy supply pathways within the metabolic power model.

Our second main finding showed that the optimization of the simulation of oxygen uptake alone did not lead to significant improvements concerning the estimation of energy supplies (Table 2). For all comparisons and variables within each exercise, there were statistically significant differences (*p* ≤ 0.003) with large effect sizes. Still, Figure 2 shows that the optimized approaches clearly follow more physiological time courses [30] compared to the metabolic power model, especially concerning the too steep descends during the repeated accelerations and sprints. Nevertheless, a potential reduction in discrepancies is conceivable, especially when considering the previously discussed MAEs of the two optimized models. However, as the original metabolic power model assumes that the metabolic power reflects the total energy expenditure, a correction of the simulation of oxygen uptake can only lead to a redistribution of aerobic–anaerobic energy supply. Even though this improves the relative distribution of supplies (Figure 3), absolute total energy expenditure remains unchanged and thus largely underestimated (Table 2). Therefore, the calculation of the aerobic energy supply by the metabolic power model must be taken into account.

In fact, the latter requirement is shown in our third main finding, where the aerobic energy supply is calculated based on the complete integral below the time course of the simulated oxygen uptake independent of the course of the metabolic power. This corrected calculation combined with the optimized simulations of oxygen uptake (Offset and Mongin model) resulted in the differences in aerobic energy supply and total energy expenditure no longer being statistically significant (*p* ≥ 0.056; trivial to large effect sizes), with the exception of the repeated sprints (*p* ≤ 0.040; moderate to large effects sizes) and total energy expenditure in the overall assessment for the Offset model (*p* = 0.006; moderate effect size) (Table 2). In this regard, the Mongin model generally performed better than the Offset model. The optimization of the simulated oxygen uptake was an essential first step, as for the original simulation by the metabolic power model, there were still statistically significant differences for all variables during all exercises (*p* ≤ 0.027; small to large effect sizes), with the exception of total energy expenditure during the continuous shuttle runs (*p* = 1.000; large effect size). Originally, the metabolic power model assumes the proportions below the time course of the simulated oxygen uptake while simultaneously being above the course of the metabolic power to serve as the oxygen debt paid and thus not being part of the aerobic energy supply [11]. However, as seen in Table 2, including that proportion into the calculation of the aerobic energy supply leads to more plausible results in comparison to the 3-component model. This indicates that, when estimating the overall energy expenditure of an exercise (i.e., of the whole body)—not that of running only, for which the metabolic power model was originally developed [6,16]—the corrected calculation is a clearly better fit. The improvements concerning the distribution of the aerobic and anaerobic energy supply in the overall assessment are also visible in Figure 3. Even though the percentage distribution of the aerobic and anaerobic energy supply suggests that the Offset model is closer to that of the 3-component model, the absolute data is more decisive for validation purposes. However, even though our approaches lead to more valid results concerning the aerobic energy supply and thus total energy expenditure, the anaerobic energy supply is still largely overestimated compared to the 3-component model—as particularly seen during the repeated sprints (Table 2, Figure 3). Since the metabolic power model defines the anaerobic energy supply by the time course of the metabolic power when above that of the simulated oxygen uptake, future studies should focus on the calculation of the metabolic power itself. Particularly given that the calculations within the metabolic power model are based on the assumption that the relative energy cost is independent of speed [5,6,16], which, however, cannot be generalized. The assumption was made for constant running, whereas the energy costs of shuttle running are up to 50% larger and increase with increasing speed [9], providing a further framework to optimize the entire metabolic power model. From a practical point of view, more research is required to clarify whether our observed differences in kJ are practically meaningful in real-world load monitoring.

Even though our study showed significant improvements concerning the discrepancies of the metabolic power model compared to the 3-component model, few limitations should be acknowledged. First, we only included twelve male intermittent team sport athletes during controlled conditions rather than in real match-play context. While the sample size may have had an impact on the statistical power, we applied a repeated measure design and collected 48 datasets, including ISRT, in total. Furthermore, our results cannot necessarily be applied to other subpopulations concerning different sexes, ages, and performance levels. This, however, is an opportunity for future studies to gain a broader understanding of different demographics. Second, we used the 3-component model as an established comparative standard in exercise science which has its own limitations. While its reliability in calculating aerobic–anaerobic energy supplies has been investigated (CV = 3.62–14.85%) [31], its validity is still being discussed. Regarding the aerobic and anaerobic alactic energy supply, both rely on the measurements by metabolic carts. Depending on the used device, accuracy in measuring, e.g., oxygen uptake (1.1–13.3%) and total energy expenditure (0.59–12.1%), can vary significantly [20]. The relative errors of the Metamax3B used in this study were 1.6% and 2.3% for oxygen uptake and total energy expenditure, respectively [20]. Additionally, the anaerobic alactic supply is based on the fast component of the excess post-exercise oxygen consumption. It is still questionable how the mathematical fitting of this phase should be conducted [32]. As for the anaerobic lactic supply, a lactate equivalent of 3 mL O_2_/kg is widely used and accepted but it is still not clear if its use is suitable for all individuals equally [25,33]. Lastly, the metabolic power model depends on external (mechanical) load measures to perform metabolic calculations. For this, valid measurements of the necessary variables, namely speed and acceleration, need to be provided. Generally, global positioning systems with higher sampling rates perform better compared to those with lower sampling rates, but are still outperformed by radar or laser technologies for acceleration measures [22]. The typical errors for the GPEXE used in our study were reported as 2.3% for speed and up to 5.6% for acceleration measures [22], being within the error rates of common metabolic cart devices.

## 5. Conclusions

In conclusion, our results show that both optimization approaches in combination with the corrected calculation of the aerobic energy supply reduce discrepancies of the tracking-based metabolic power model for estimating energy expenditure and aerobic–anaerobic supplies during intermittent running compared to the established 3-component model. However, the anaerobic energy supply is still largely overestimated and should be treated with caution. Future research should further improve the model by integrating heart rate data and reconsidering the calculation of the metabolic power model itself, e.g., filtering techniques for speed and acceleration, equivalent slope approach, and energy cost of constant running.

## Figures and Tables

**Table 1:** Prediction accuracy of the simulated oxygen uptake by the original metabolic power model, the optimized Offset, and Mongin model compared to the measured oxygen uptake (n = 12).

Exercise	MPM Mean ± SD		Offset Mean ± SD		Mongin Mean ± SD	
	RMSE (mL/min/kg)	MAE (mL/min/kg)	RMSE (mL/min/kg)	MAE (mL/min/kg)	RMSE (mL/min/kg)	MAE (mL/min/kg)
Continuous shuttle runs	5.42 ± 2.59	4.87 ± 2.71	4.19 ± 1.82	3.60 ± 1.92	5.05 ± 2.30	4.51 ± 2.42
Repeated accelerations with COD	6.91 ± 1.54	5.88 ± 1.48	4.71 ± 0.98	3.82 ± 0.76	4.71 ± 1.04	3.88 ± 0.83
Repeated sprints with COD	13.49 ± 1.08	11.71 ± 0.91	10.24 ± 0.92	8.32 ± 0.77	8.37 ± 1.71	6.58 ± 1.65
Overall	8.61 ± 1.13	7.49 ± 1.13	6.38 ± 0.70	5.25 ± 0.70	6.05 ± 1.10	4.99 ± 1.12

Means and standard deviations are shown. COD = change of direction; MAE = mean absolute error; MPM = metabolic power model; RMSE = root mean square error.

**Table 2:** Differences in total energy expenditure and energy supplies of the three running-based exercises using the 3-component and metabolic power model as well as the optimized Offset and Mongin model (n = 12).

Exercise	Variables	3-CM Mean ± SD	MPM Mean ± SD	Offset Mean ± SD	Mongin Mean ± SD	Global *p*-Value	3-CM vs. MPM	3-CM vs. Offset	3-CM vs. Mongin
Continuous shuttle runs	W_ANA_ (kJ) ^b^	5.6 ± 2.1	92.1 ± 16.0	46.8 ± 13.3	72.0 ± 42.7	<0.001 ^large^	0.003 ^large^	0.003 ^large^	0.003 ^large^
	W_AER_ (kJ) ^a^	483.0 ± 75.7	313.0 ± 59.4	258.2 ± 63.4	333.0 ± 44.6	<0.001 ^large^	<0.001 ^large^	<0.001 ^large^	<0.001 ^large^
	W_AER__COR (kJ) ^a^		390.7 ± 71.4	489.2 ± 71.3	442.1 ± 42.1	<0.001 ^large^	<0.001 ^large^	1.000 ^trivial^	0.840 ^moderate^
	W_TOT_ (kJ) ^a^	488.7 ± 76.1	405.1 ± 74.0	405.1 ± 74.0	405.1 ± 74.0	<0.001 ^large^	0.002 ^large^	0.002 ^large^	0.002 ^large^
	W_TOT__COR (kJ) ^a^		482.7 ± 86.5	536.0 ± 82.7	514.1 ± 60.3	0.009 ^small^	1.000 ^large^	0.067 ^moderate^	1.000 ^small^
Repeated accelerations with COD	W_ANA_ (kJ) ^b^	4.6 ± 1.9	46.8 ± 10.5	40.2 ± 9.8	43.4 ± 11.8	<0.001 ^large^	0.003 ^large^	0.003 ^large^	0.003 ^large^
	W_AER_ (kJ) ^a^	188.8 ± 61.8	40.1 ± 14.3	46.8 ± 15.1	43.6 ± 13.3	<0.001 ^large^	0.003 ^large^	0.003 ^large^	0.003 ^large^
	W_AER__COR (kJ) ^a^		84.1 ± 24.2	185.8 ± 23.8	177.1 ± 13.8	<0.001 ^large^	0.003 ^large^	1.000 ^trivial^	1.000 ^small^
	W_TOT_ (kJ) ^a^	193.4 ± 63.3	86.9 ± 23.9	86.9 ± 23.9	86.9 ± 23.9	<0.001 ^large^	0.003 ^large^	0.003 ^large^	0.003 ^large^
	W_TOT__COR (kJ) ^a^		130.9 ± 34.3	226.0 ± 33.2	220.5 ± 22.8	<0.001 ^large^	0.003 ^large^	0.097 ^moderate^	0.463 ^moderate^
Repeated sprints with COD	W_ANA_ (kJ) ^a^	68.3 ± 14.7	218.6 ± 38.7	202.7 ± 38.5	223.0 ± 49.9	<0.001 ^large^	0.003 ^large^	0.003 ^large^	0.003 ^large^
	W_AER_ (kJ) ^b^	518.3 ± 64.7	144.7 ± 25.1	160.6 ± 25.5	140.3 ± 14.6	<0.001 ^large^	0.003 ^large^	0.003 ^large^	0.003 ^large^
	W_AER__COR (kJ) ^a^		331.2 ± 52.1	435.5 ± 51.9	400.8 ± 24.5	<0.001 ^large^	<0.001 ^large^	<0.001 ^large^	<0.001 ^large^
	W_TOT_ (kJ) ^a^	586.6 ± 74.2	363.3 ± 61.6	363.3 ± 61.6	363.3 ± 61.6	<0.001 ^large^	<0.001 ^large^	<0.001 ^large^	<0.001 ^large^
	W_TOT__COR (kJ) ^a^		549.8 ± 89.7	638.2 ± 89.1	623.8 ± 59.3	<0.001 ^large^	0.027 ^small^	0.002 ^moderate^	0.040 ^moderate^
Overall	W_ANA_ (kJ) ^b^	78.5 ± 16.8	357.5 ± 59.3	289.7 ± 56.3	338.4 ± 100.0	<0.001 ^large^	0.003 ^large^	0.003 ^large^	0.003 ^large^
	W_AER_ (kJ) ^b^	1190.2 ± 185.9	497.8 ± 91.2	565.6 ± 95.7	516.9 ± 60.6	<0.001 ^large^	0.003 ^large^	0.003 ^large^	0.003 ^large^
	W_AER__COR (kJ) ^b^		806.0 ± 138.0	1110.6 ± 137.5	1020.0 ± 63.3	<0.001 ^large^	0.003 ^large^	0.056 ^small^	0.126 ^large^
	W_TOT_ (kJ) ^b^	1268.7 ± 196.5	855.3 ± 148.0	855.3 ± 148.0	855.3 ± 148.0	<0.001 ^large^	0.003 ^large^	0.003 ^large^	0.003 ^large^
	W_TOT__COR (kJ) ^b^		1163.5 ± 195.8	1400.3 ± 191.3	1358.4 ± 123.2	<0.001 ^large^	0.006 ^moderate^	0.006 ^moderate^	0.384 ^moderate^

Means, standard deviations, *p*-values, and interpretations of effect sizes (superscripted) are shown. 3-CM = 3-component model; COD = change of direction; MPM = metabolic power model; W_AER_ = aerobic energy supply; W_AER__COR = corrected aerobic calculation; W_ANA_ = anaerobic energy supply; W_TOT_ = total energy expenditure; W_TOT__COR = total energy expenditure with W_AER__COR. ^a^ analyzed by one-way repeated measures ANOVA and *t*-test. ^b^ analyzed by Friedman and Wilcoxon test.

## Data Availability

All data is included in this article and the Supplementary Materials.

## Supplementary Materials

The following supporting information can be downloaded at https://www.mdpi.com/article/10.3390/s25247568/s1, Data S1.

## Abbreviations

The following abbreviations are used in this manuscript:

ISRT	Interval shuttle run test
MAE	Mean absolute error
RMSE	Root mean square error
